# *Piper betle* leaf extract affects the quorum sensing and hence virulence of *Pseudomonas aeruginosa* PAO1

**DOI:** 10.1007/s13205-015-0348-8

**Published:** 2016-01-09

**Authors:** Siraj Datta, Debanjan Jana, Tilak Raj Maity, Aveek Samanta, Rajarshi Banerjee

**Affiliations:** Department of Biotechnology, Haldia Institute of Technology, Haldia, West Bengal 721657 India

**Keywords:** Anti Quorum Sensing properties, *Piper betle* leaf extract, *Pseudomonas aeruginosa*, Biofilm production, Pyocyanin assay, Mobility pattern

## Abstract

Quorum sensing (QS) plays an important role in virulence of *Pseudomonas aeruginosa*, blocking of QS ability are viewed as viable antimicrobial chemotherapy and which may prove to be a safe anti-virulent drug. Bioactive components from *Piper betle* have been reported to possess antimicrobial ability. This study envisages on the anti-QS properties of ethanolic extract of *P. betle* leaf (PbLE) using *P. aeruginosa* PAO1 as a model organism. A marked reduction in swarming, swimming, and twitching ability of the bacteria is demonstrated in presence of PbLE. The biofilm and pyocyanin production also shows a marked reduction in presence of PbLE, though it does not affect the bacterial growth. Thus, the studies hint on the possible effect of the bioactive components of PbLE on reducing the virulent ability of the bacteria; identification of bioactive compounds should be investigated further.

**Electronic supplementary material:**

The online version of this article (doi:10.1007/s13205-015-0348-8) contains supplementary material, which is available to authorized users.

## Introduction

Amongst proteobacteria, a widespread cell-to-cell communication (quorum sensing; QS) phenomenon is observed (Krishnan et al. 2012). QS is used to regulate the diverse bacterial function amongst which bioluminescence, biofilm formation, virulence, pigment production, motility and polysaccharide production have been widely studied (Fuqua and Greenberg 2002; Whitehead et al. 2001). *Pseudomonas aeruginosa* (belonging to Gamma Proteobacteria) is a prevalent opportunistic human pathogen and primarily infecting immune compromised patients (Govan and Deretic 1996). It causes serious eye (Zhu et al. 2004), ear (Tron et al. 2004), burn wounds (Friedstat et al. 2013), urinary tract (Packiavathy et al. 2014) and respiratory tract infection (cystic fibrosis) (Smith et al. 2013; Lyczak et al. 2002). Available antibiotic therapy does not respond to these infections; especially predisposed to infection with *P. aeruginosa* and hence the bacterium is developing new resistance, responsible for high rates of morbidity and mortality (Lanini et al. 2011). Alternative strategies to conventional antibiotic therapy are therefore required.

QS helps the bacteria to detect their population density by producing, releasing and perceiving the small autoinducer molecules and coordinate a common action such as releasing the virulence factors (Girard and Bloemberg 2008; Jimenez et al. 2012; Kumar et al. 2015). Thus, the virulence phenotypes of the bacteria can be quenched by blocking the QS. QS inhibitor (QSI) may inhibit the QS mechanism and be able to attenuate the virulence of the pathogen and are helpful to break the antibiotic resistance (Vattem et al. 2007; Adonizio et al. 2008). Recent studies have been demonstrated that QSI compound(s) can be found in higher plants such as vanilla (Choo et al. 2006), raspberry (Vattem et al. 2007), clove (Krishnann et al. 2012). In light of these findings, we look into Ayurveda, the oldest traditional medicine system of India, which reports large number of herbs possessing potential preventive and curative properties (Mukherjee and Wahile 2006). In Ayurveda, the use of betel leaves (*Piper betle* L.) in various ways, as carminative, stimulant, antiseptic, antifungal, antibacterial, anti-diabetic and anti-allergic agent have been mentioned (Guha 2006). The extract of betel leaves have been reported to possess many biological activities that are able to control the growth of many Gram positive and Gram negative microbes (Nair and Chanda 2008). No information however is available on betel leaf extracts to demonstrate its anti-QS activity.

In this study, we report of the anti-QS properties of ethanolic extract *P. betle* leaf (PbLE); effects of the mobility patterns (namely Swarming, Swimming and Twitching), reduction on biofilm and pyocyanin production in presence of different concentrations of PbLE. This in turn reflects on the virulence of model microorganism *P. aeruginosa* PAO1.

## Materials and methods

### Bacterial strains and subculture conditions

*Pseudomonas aeruginosa* PAO1 (MTCC-3541) was obtained from microbial type culture collection and Gene Bank, Chandigarh, India. The stock culture of the test organism was maintained in medium containing 30 % glycerol in cryogenic vials were kept at −70 °C. Working cultures were kept at 4 °C on nutrient agar slants and were periodically transferred to fresh slants. A loop full of culture from the slants were transferred to nutrient broth and grown overnight at 37 °C. The overnight grown culture was used for the subsequent study.

### Preparation of ethanolic extract of betel leaves (PbLE)

Freshly cut betel leaves (*Piper betle* L. ver. Kali Bangla; landrace of Paschim Medinipur, West Bengal, India) were dried in hot air oven (40 ± 1 °C) for 48 h, crushed in mortar and pestle and the leaf powder were stored at −20 °C. Leaf powder was Soxhlet extracted using 80 % ethanol for 20 h. The crude extract was concentrated and dried in rotary vacuum evaporator below 50 °C, 100 mg dried extract was dissolved in 500 µl of dimethylsulfoxide (DMSO) and then diluted to 10 mg/ml working PbLE stock by adding triple distilled water (Maity et al. 2014).

### Mobility patterns assays

#### Swarming assay

Tan et al. (2013) was followed with minor modifications for the preparation of the swarm plate assay of *P. aeruginosa* PAO1. Petri plates were prepared using 0.5 g bacto agar, 0.5 g peptone, 0.2 g yeast extract and 1.0 g glucose per 100 ml of distilled water. A set containing 25, 50, 75, 100 µg/ml of PbLE was seeded with 5 ml of media and poured immediately on a 10 ml of pre-warmed agar plate as an overlay. Overnight culture of the *P. aeruginosa* PAO1 culture was inoculated at the centre of the agar surface and the plate was incubated for 24 h at 37 °C.

With minor modifications, swimming and twitching assay was done following Inoue et al. (2008).

#### Swimming assay

Like the swarming plate, swimming plate assay media contained 1 % nutrient broth, glucose 0.5 %, agar 0.3 %. The agar media was air dried for 5–10 min and the bacterial cells were gently inoculated using a tooth pick at the centre of the agar surface. Then the plate was incubated at 37 °C for 24–48 h.

#### Twitching assay

The media for twitching assay contained 1 % tryptone, 0.5 % yeast extract, 0.5 % NaCl, 1 % agar. In twitching assay, bacterial cells were stabbed into the bottom of a petri dish containing the said agar medium using a toothpick and incubated at 37 °C for 20 h. The movement of the colony on the interface between the agar medium and the petri dish was observed.

### Pyocyanin assay

Pyocyanin was extracted from overnight grown *P. aeruginosa* PAO1 culture supernatant. 25 µg/ml up to 200 µg/ml (25, 50, 75, 100, 125, 150, 175 and 200 µg/ml) of PbLE were mixed with freshly prepared *P. aeruginosa* culture (2 ml, OD_600_ = 0.1) and incubated overnight at 37 °C. After 24 h, 2 ml of chloroform was added to the culture supernatant and mixed vigorously. The chloroform layer was mixed with 1 ml of HCl (0.2 M). After centrifugation (8000 rpm for 10 min at 28 °C) the relative concentration of Pyocyanin was measured as OD of the HCl layer at 520 nm against 0.2 M HCl as blank (Chong et al. 2011).

### Biofilm formation and quantification

An overnight culture of *P. aeruginosa* PAO1 was diluted to 1:100 into fresh medium and 100 µl was added to each well of microtiter plate and incubated for 28–30 h at 37 °C. After incubation, cells were dumped out by turning the plate over and shaking out the liquid. The plates were submerged in small tub of water, and shake the water out. The process was repeated. 125 µl of a 0.1 % aqueous solution of a crystal violet was added and the plates are incubated at room temperature for 10–15 min. Plates were rinsed 3–4 times in water and blotted on a stack of paper towels by turning the plates upside down and dried for a few hours or overnight. To quantitate, 200 µl of 30 % acetic acid was added to each well. Incubated for 10–15 min and 125 µl was transferred to a new, flat-bottomed microtiter dish. OD at A_540_–A_600_ was read on a plate reader (Siddiqui et al. 2012).

### Assessment of bacterial growth

Actively growing culture of *P. aeruginosa* PAO1 (1 ml, OD_600_ = 0.1) was incubated in Luria Broth with different concentrations of PbLE (50, 100, 150 µg/ml). OD_600_ nm was observed at every 2 h interval for consecutively 40 h to evaluate the relative growth in different experimental conditions.

### Statistical analysis

All the above experiments were carried out in triplicates. The data were statistically analysed by conducting student’s *t* test and ANOVA. Statistical analysis and the graphs were constructed using MS excel.

## Results and discussion

### Mobility patterns: swarming, swimming and twitching

**Fig. 1:**
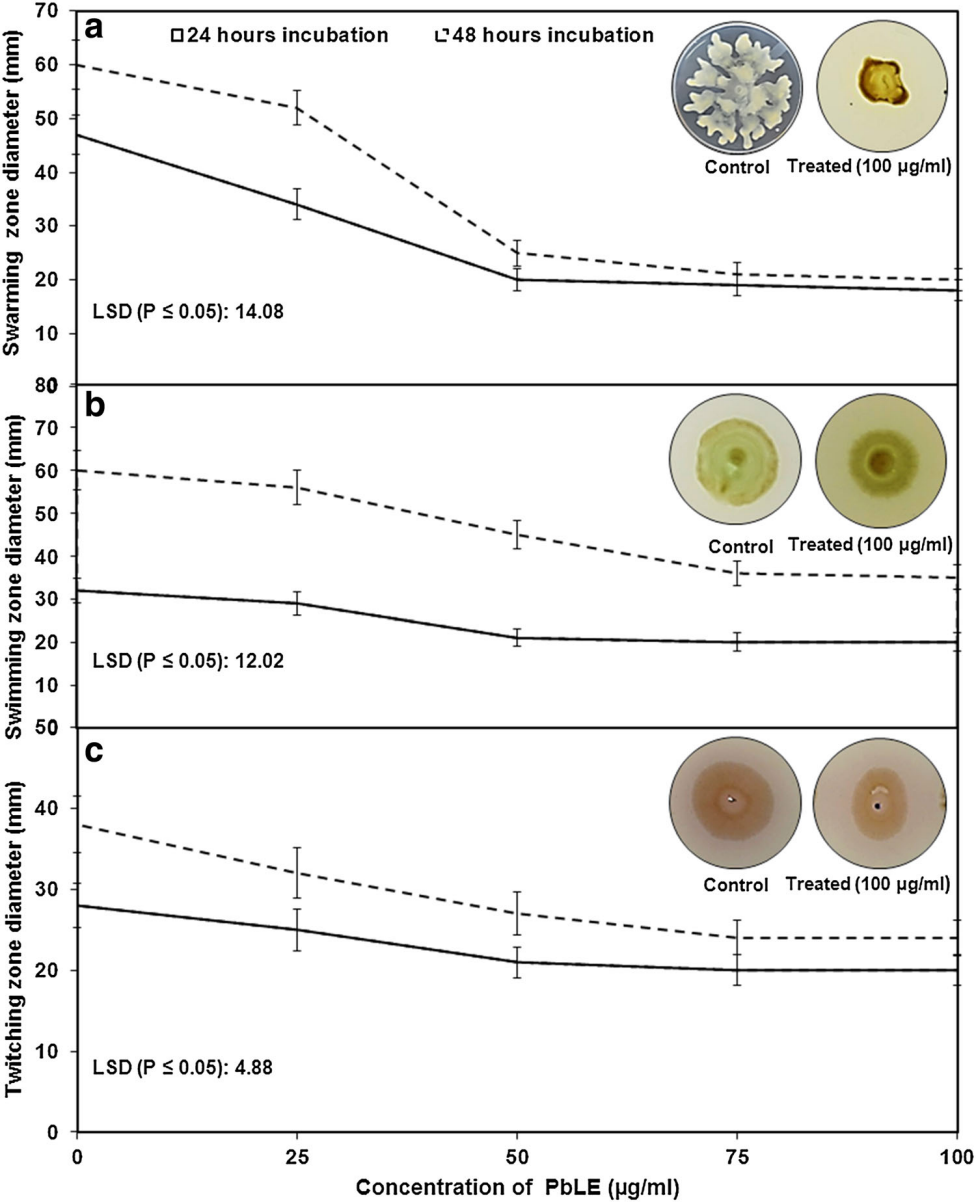
Effect of *piper betle* ethanolic leaf extract (PbLE) on the mobility pattern of *Pseudomonas aeruginosa* PAO1. **a** Effect of swarming activity, **b** swimming activity and **c** twitching activity of *Pseudomonas aeruginosa* PAO1 with increasing concentrations (25, 50, 75 and 100 μg/ml) of PbLE (*inset* showing the colony morphology of the bacteria)

The graph (Fig. 1a) shows that a concentration beyond 25 μg/ml PbLE inhibits the swarming activity drastically. A PbLE concentration of 50 μg/ml was seen to be inhibitory for swarming activity of the bacteria wherein more than 50 % inhibition was observed irrespective of the hours of incubation. The slope between 25 and 50 μg/ml (*m* = 0.56) after 24 h while the slope at the same concentration after 48 h (*m* = −1.08) indicates significantly that the rate of inhibition drastically increases after 24 h.

After 24 h of incubation at 25 μg/ml with respect to control a difference of 13 mm in the zone of inhibition are observed. At the same period of incubation at 50 μg/ml swimming activity of *P. aeruginosa* PAO1 in presence of PbLE (Fig. 1b) diminished with increase in concentration (at 24 h) but after 48 h of exposure, the rate of inhibition is almost 7.40 %. Though, beyond 50 μg/ml the inhibition rate comes to a constant irrespective of the hours of incubation.

Twitching activity in presence of PbLE (Fig. 1c) was seen to be diminishing but is not much of significance when compared to the control; a mere 10.71 % decrease is seen after 24 h of incubation at 25 μg/ml. Thereafter the rate of decrease remains constant.

Form the above observations, it could be said that PbLE has a destructive effect on the QS mechanisms of *P. aeruginosa* PAO1. The above inhibition activities were compared simultaneously with certain antibiotics like Ciprofloxacin, Gentamicin. The antibiotic Ciprofloxacin inhibited the growth of *P. aeruginosa* PAO1 but was found to be resistant to the antibiotic Gentamicin (Data not shown). Of all three QS motility mechanisms observed it has the most devastating effect on swarming activity which correlates with the findings of Tan et al. (2013).

### Biofilm and pyocyanin assay; inhibition biology of biofilm formation and resistance

**Fig. 2:**
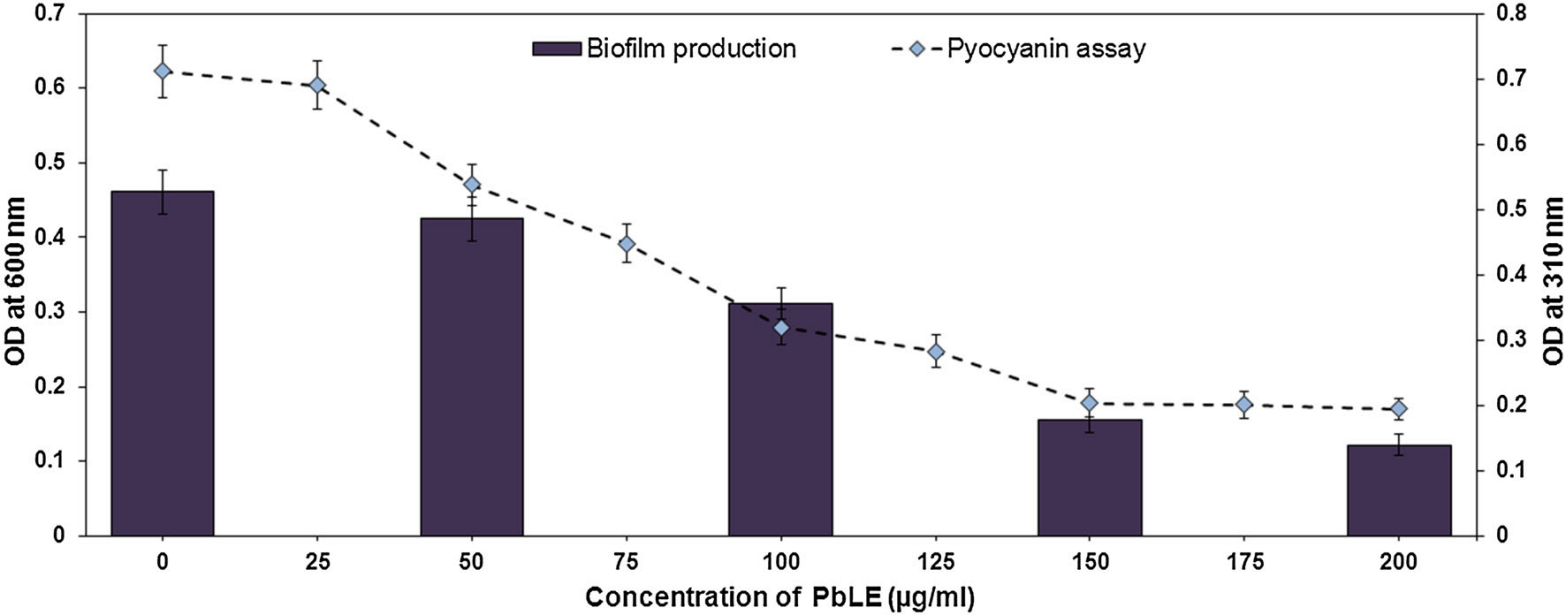
Inhibition of *Pseudomonas aeruginosa* PAO1 biofilm and pyocyanin production in presence of different concentrations of PbLE. *Bar chart* represents the mean results from triplet cultures of three independent experiments, with *error bar* representing standard deviation

In our studies, a reduction is observed in biofilm production (Fig. 2) at concentrations beyond 50 μg/ml. When compared to control sets the inhibition percentage could be said as 50 μg/ml (7.81 %), 100 μg/ml (32.54 %), 150 μg/ml (66.16 %), 200 μg/ml (75.35 %) i.e. almost approximately fourfolds of decrease per 50 μg/ml of increase in PbLE concentration. Because biofilm resistance depends on aggregation of bacteria in multicellular communities, one strategy might be to develop therapies that disrupt the multicellular structure of the biofilm. If the multi-cellularity of the biofilm is defeated, the host defences might be able to resolve the infection, and the efficacy of antibiotics might be restored. Potential therapies include enzymes that dissolve the matrix polymers of the biofilm, chemical reactions that block biofilm matrix synthesis, and analogues of microbial signalling molecules that interfere with cell-to-cell communication, required for normal biofilm formation (Stewart and Costerton 2001).

This study lays a foundation that a simultaneous administration of PbLE along with antibiotics (reported to be resistant to *P. aeruginosa*) may help in bringing back the sensitivity to effect in antibiotic therapy.

Pyocyanin contributes to the persistence of *P. aeruginosa* infection by causing detrimental effects toward lung (Lyczak et al. 2002) epithelial cells and by deregulating inflammatory response initiated by the host. Pyocyanin synthesis is regulated by a complex synchrony of lasR-lasI, rhlR-rhlI and mvfR-haq QS-system whereby mutations in these systems lead to the deficiency of pyocyanin synthesis (Priya et al. 2013).

Our studies depict Pyocyanin production to be having a steady decrease with increasing concentrations of PbLE i.e. beyond 150 μg/ml the rate of decrease comes to a constant (Fig. 2).

From our studies and available literature, it could be postulated that the PbLE may have inhibited the QS-systems at its gene expression level or acts as an antagonist to decrease pyocyanin production.

**Fig. 3:**
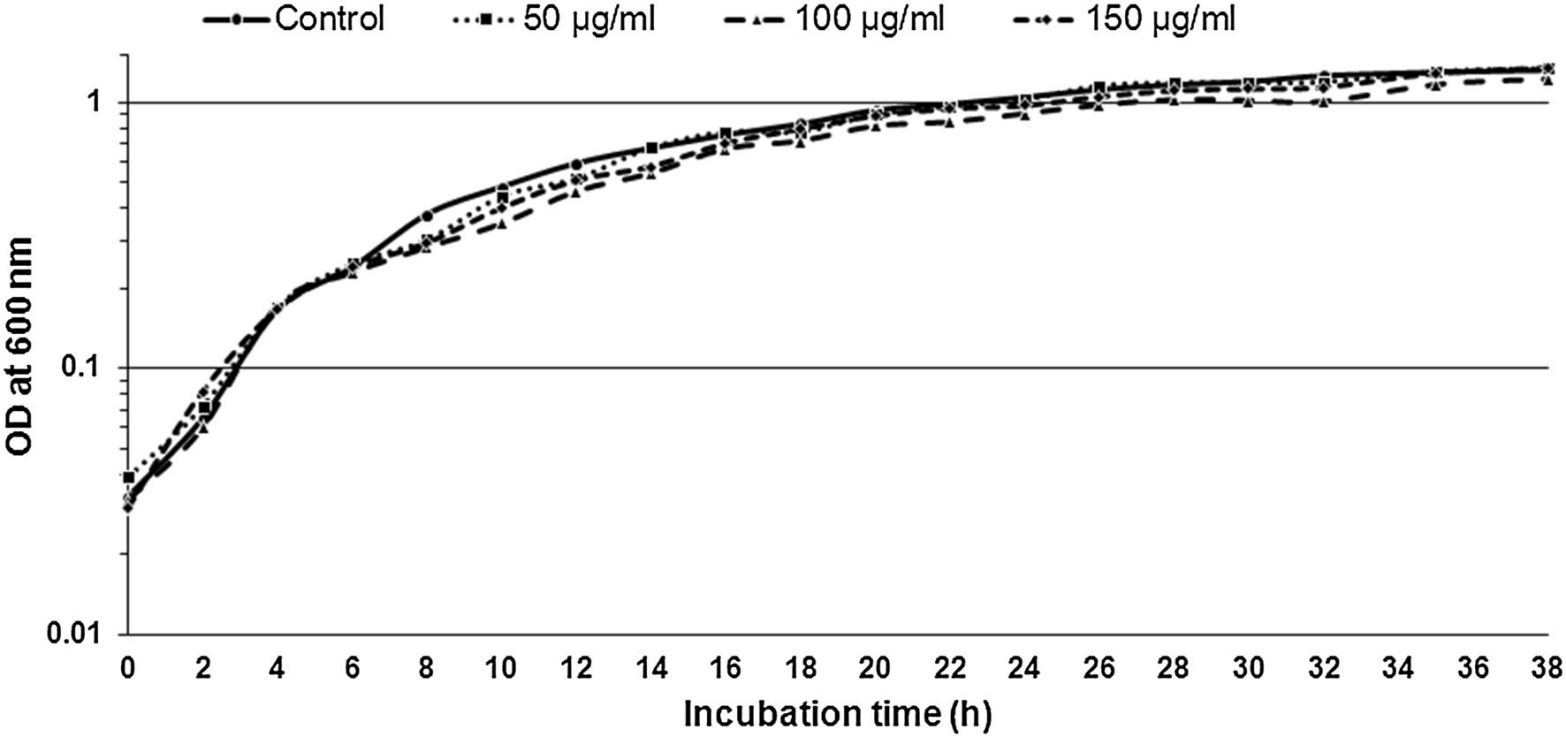
Growth curve patterns of *Pseudomonas aeruginosa* PAO1 in presence of different concentrations of PbLE (0, 50, 100 and 150 μg/ml)

Comparing the above observations (Fig. 3) with the growth curve of *P. aeruginosa* PAO1 in presence different concentrations of PbLE; it does not show any variation or decrease in the growth pattern as compared to PbLE free medium. Thus, it makes it evident that PbLE irrespective of its concentration has no effect on the cell division of *P. aeruginosa* PAO1 but the biofilm assay and pyocyanin production does indicate that PbLE has a marked effect on the anabolism of biofilm and pyocyanin production. Hence, this effect also has an effect on the QS abilities of the bacteria in presence of PbLE. This indicates on the possible reducing virulence of *P. aeruginosa*.

## Conclusion

Based on the results obtained from this study, it is proven that the ethanolic extract of *P. betle* demonstrates anti-QS capabilities. It may have also been able to attenuate QS-regulated virulence determinants of *P. aeruginosa* PAO1. Newer insights may be provided by the bioactive compounds in the extract leading towards discovering potential anti-pathogenic drugs to combat emerging multidrug resistant pathogens. Future work should direct to the isolation and characterization of active molecule that is responsible for the anti-QS properties of the ethanolic extract of *P. betle* leaf.

## Electronic supplementary material

Below is the link to the electronic supplementary material.

Supplementary material 1 (DOCX 16 kb)
