# Supplementary material for: Piper betle leaf extract affects the quorum sensing and hence virulence of Pseudomonas aeruginosa PAO1
Source: 3 Biotech. 2016 Jan 9;6(1):18. doi: 10.1007/s13205-015-0348-8 (PMC4706831; doi:10.1007/s13205-015-0348-8)
Supplement: Supplementary file 1 — Supplementary material 1 (DOCX 16 kb) [file 13205_2015_348_MOESM1_ESM.docx]

Table 1: Effect ethanolic leaf extracts of piper betle (PbLE) on the mobility pattern (Swarming activity, Swimming activity and Twitching activity) of *Pseudomonas aeruginosa* PAO1 with increasing concentration (25, 50, 75 and 100 μg/ml).

| **Incubation time** | **Swarming zone diameter (mm)** | | | | |  | **Swimming zone diameter (mm)** | | | | |  | **Twitching zone diameter (mm)** | | | | |
| --- | --- | --- | --- | --- | --- | --- | --- | --- | --- | --- | --- | --- | --- | --- | --- | --- | --- |
|  | **Concentration of PbLE (µg/ml)** | | | | |  | **Concentration of PbLE (µg/ml)** | | | | |  | **Concentration of PbLE (µg/ml)** | | | | |
|  | **0** | **25** | **50** | **75** | **100** |  | **0** | **25** | **50** | **75** | **100** |  | **0** | **25** | **50** | **75** | **100** |
| **24 h** | 47±3.7 | 34±2.9 | 20±2.1 | 19±2 | 18±1.9 |  | 32±2.8 | 29±2.7 | 21±2 | 20±2.1 | 20±2.1 |  | 28±2.7 | 25±2.6 | 21±1.9 | 20±1.9 | 20±1.8 |
| **48 h** | 60±4.6 | 52±3.2 | 25±2.4 | 21±2.1 | 20±2 |  | 60±4.5 | 56±3.9 | 45±3.3 | 36±2.9 | 35±2.9 |  | 38±3.5 | 32±3.1 | 27±2.6 | 24±2.1 | 24±2.1 |
| **Mean** | 53.5 | 43 | 22.5 | 20 | 19 |  | 46 | 42.5 | 33 | 28 | 27.5 |  | 33 | 28.5 | 24 | 22 | 22 |
| **LSD (P ≤ 0.05)** | 14.08 | | | | |  | 12.02 | | | | |  | 4.88 | | | | |
